# IL11 modulates MMP12 expression and cancer cell metastasis via IL11RA/IL6ST-PI3K/Akt/NF-κB pathway in lung cancer

**DOI:** 10.1038/s41420-026-03163-2

**Published:** 2026-06-11

**Authors:** Chiang-Wen Lee, Shih-Sen Lin, Tsung-Ming Chang, Zih-Chan Lin, Yao-Chang Chiang, Miao-Ching Chi, Kuo-Ti Peng, Mei-Ling Fang, Ju-Fang Liu

**Affiliations:** 1 Department of Respiratory Care, Chang Gung University of Science and Technology, Chiayi, Taiwan, ROC; 2 Chronic Diseases and Health Promotion Research Center and Center for smart healthcare education and Drug Research and Development, Chang Gung University of Science and Technology, Chiayi, Taiwan, ROC; 3 Department of Orthopaedic Surgery, Chang Gung Memorial Hospital, Chiayi, Taiwan, ROC; 4 Department of Safety Health and Environmental Engineering, Ming Chi University of Technology, New Taipei City, Taiwan, ROC; 5 Division of Chest Medicine, Department of Internal Medicine, Shin Kong Wu Ho-Su Memorial Hospital, Taipei, Taiwan, ROC; 6 School of Dental Technology, College of Oral Medicine, Taipei Medical University, Taipei, Taiwan, ROC; 7 Division of Pulmonary and Critical Care Medicine, Chang Gung Memorial Hospital, Chiayi, Taiwan, ROC; 8 College of Medicine, Chang Gung University, Taoyuan, Taiwan, ROC; 9 Center for Environmental Toxin and Emerging-Contaminant Research, Cheng Shiu University, Kaohsiung, Taiwan, ROC; 10 Department of Civil Engineering and Geomatics, Cheng Shiu University, Kaohsiung, Taiwan, ROC; 11 Translational Medicine Center, Shin-Kong Wu Ho-Su Memorial Hospital, Taipei, Taiwan, ROC; 12 School of Oral Hygiene, College of Oral Medicine, Taipei Medical University, Taipei, Taiwan, ROC; 13 Department of Medical Research, China Medical University Hospital, China Medical University, Taichung, Taiwan, ROC

**Keywords:** Non-small-cell lung cancer, Extracellular matrix

## Abstract

Lung cancer is a leading cause of cancer-related mortality, with metastasis significantly reducing patient survival. Interleukin 11 (IL11), a member of the IL-6 cytokine family, has been associated with cancer progression, yet its role in non-small cell lung cancer (NSCLC) metastasis remains unclear. This study analyzed public datasets and demonstrated that IL11 is upregulated in NSCLC and correlates with lymphatic metastasis and poor prognosis. Functional assays revealed that IL11 enhances lung cancer cell migration through upregulation of matrix metalloproteinase 12 (MMP12). Mechanistically, IL11 acts via the IL11 receptor subunit alpha (IL11RA)/ IL6 cytokine family signal transducer (IL6ST) complex to activate the PI3K/Akt/NF-κB signaling pathway, which in turn drives MMP12 expression and promotes metastatic behavior. Notably, the clinically approved selective estrogen receptor modulator bazedoxifene effectively inhibited IL11-induced signaling, reduced MMP12 levels, and suppressed cancer cell migration in vitro. In an orthotopic lung cancer mouse model, IL11 knockdown significantly reduced tumor growth and intrapulmonary spread, accompanied by decreased IL11, MMP12, and phosphorylated NF-κB p65 levels in lung tissues. These findings uncover a novel IL11-driven pathway contributing to metastatic behavior in NSCLC and identify the IL11/IL11RA/IL6ST axis as a potential therapeutic target.

**Figure:**
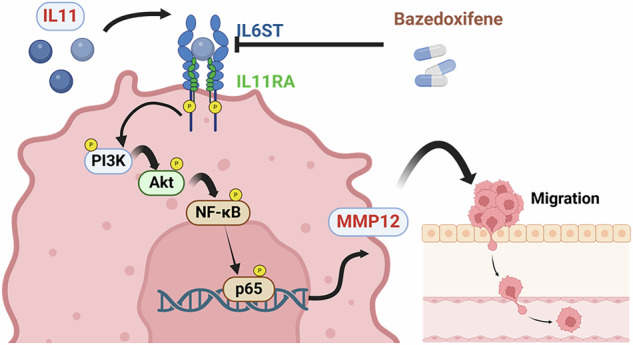
Schematic representation of bazedoxifene-mediated suppression of IL11-induced pro-metastatic signaling in lung cancer. Non-small cell lung cancer (NSCLC) cells secrete IL11, which binds to the IL11RA/IL6ST receptor complex and activates the PI3K/Akt signaling pathway. This activation enhances NF-κB transcriptional activity, leading to upregulated MMP12 expression and promoting lung cancer cell migration. Notably, bazedoxifene effectively blocks IL11-induced PI3K/Akt activation, thereby suppressing NF-κB signaling and reducing MMP12 expression, which ultimately attenuates NSCLC metastasis.

Schematic representation of bazedoxifene-mediated suppression of IL11-induced pro-metastatic signaling in lung cancer. Non-small cell lung cancer (NSCLC) cells secrete IL11, which binds to the IL11RA/IL6ST receptor complex and activates the PI3K/Akt signaling pathway. This activation enhances NF-κB transcriptional activity, leading to upregulated MMP12 expression and promoting lung cancer cell migration. Notably, bazedoxifene effectively blocks IL11-induced PI3K/Akt activation, thereby suppressing NF-κB signaling and reducing MMP12 expression, which ultimately attenuates NSCLC metastasis.

## Introduction

Lung cancer is a leading cause of cancer-related death worldwide [1]. Among its types, non-small cell lung cancer (NSCLC) accounts for approximately 85% of cases [2]. Metastasis significantly worsens prognosis, reducing the five-year survival rate to around 4% [3, 4]. Conventional therapies are often ineffective once metastasis has occurred, highlighting the need to explore molecular mechanisms driving NSCLC metastasis [5, 6].

Tumor metastasis involves multiple steps, including detachment, invasion, circulation, and colonization of distant sites [7]. The tumor microenvironment, particularly extracellular matrix (ECM) components and cytokines, is crucial in this process and is increasingly recognized as a major determinant of NSCLC progression, metastatic dissemination, and therapeutic response [8, 9]. Matrix metalloproteinases (MMPs) play a key role by degrading ECM and promoting invasion [10, 11]. However, how specific cytokines regulate MMP expression in NSCLC remains underexplored.

Interleukin 11 (IL11), a member of the IL-6 cytokine family, is involved in inflammation, tissue repair, and tumor progression [12]. Elevated IL11 levels have been linked to metastasis in various cancers. In NSCLC, IL11 is upregulated and correlates with advanced disease and poor prognosis [13, 14]. It is known to promote epithelial-mesenchymal transition (EMT) and may regulate metastasis through IL11 receptor subunit alpha (IL11RA)/IL6 cytokine family signal transducer (IL6ST) receptor signaling [15–17]. Additionally, IL11 signaling has been implicated in the progression and metastasis of multiple cancers, including intrahepatic cholangiocarcinoma, pancreatic, bladder, prostate, and gastric cancers [18–22]. In NSCLC, elevated IL11 levels in patient serum and lung cancer cell-derived microvesicles correlate with increased metastatic severity [23, 24]. Moreover, recombinant human IL11 treatment significantly enhances proliferation and induces EMT in A549 cells [25]. Recent evidence further supports a pathogenic role of the IL-11/IL-11RA axis in LUAD, showing its association with poor clinical outcomes and demonstrating that therapeutic targeting of IL-11RA exerts anti-tumor activity in vivo [26]. Despite these findings, the precise role of IL11 in lung cancer metastasis remains to be fully elucidated.

Bazedoxifene is a third-generation selective estrogen receptor modulator (SERM) initially developed for postmenopausal osteoporosis prevention. It exerts tissue-specific estrogenic and anti-estrogenic effects, making it effective in hormone-related conditions [27]. Beyond its established clinical use, recent studies have identified bazedoxifene as an inhibitor of IL-6/IL6ST signaling, a pathway implicated in cancer progression and metastasis [28, 29]. Bazedoxifene has demonstrated promising anti-tumor activity in various malignancies, including breast and pancreatic cancers [29]. In NSCLC, bazedoxifene has been reported to reduce cell viability, colony formation, and migration while enhancing the efficacy of chemotherapy agents such as paclitaxel and gemcitabine [30]. However, whether IL11 plays a direct role in NSCLC metastasis and whether bazedoxifene can effectively inhibit IL11-induced metastasis remains unclear. Addressing these gaps could reveal new therapeutic opportunities for lung cancer treatment.

Here, we investigated the mechanistic role of IL11 in NSCLC progression, with a particular focus on its ability to regulate MMP12 expression and metastatic behavior through the IL11RA/IL6ST-PI3K/Akt-NF-κB signaling axis. We further examined whether pharmacological inhibition with bazedoxifene could suppress IL11-induced signaling and cell motility in vitro, and whether IL11 knockdown could attenuate tumor progression and intrapulmonary spread in an orthotopic lung cancer model in vivo.

## Results

### IL11 is highly expressed in NSCLC and correlates with the metastatic stage

**Fig. 1:**
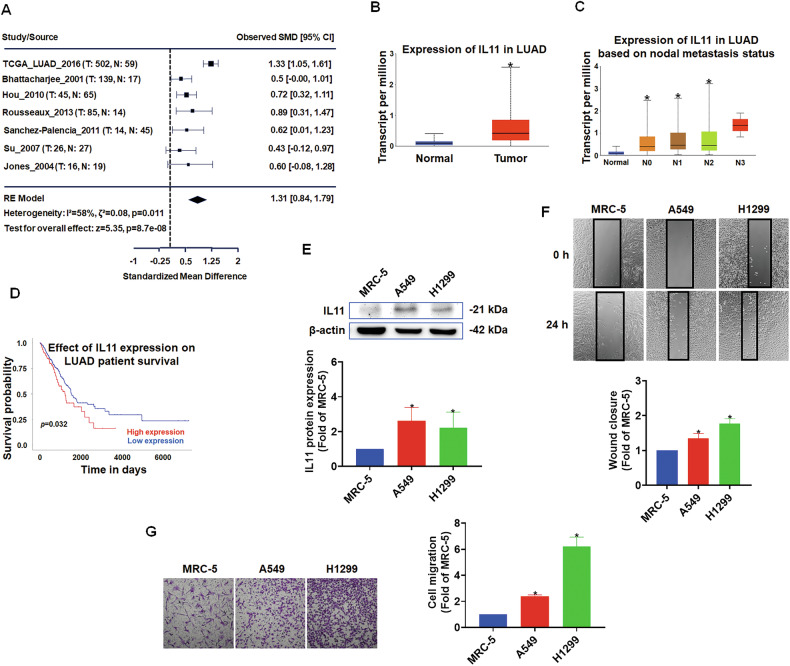
IL11 is overexpressed in lung cancer. **A** Meta-analysis of IL11 expression in normal and lung adenocarcinoma (LUAD) tissues using data from the Lung Cancer Explorer (LCE). **B**, **C** IL11 gene expression in normal lung tissue and LUAD obtained from The Cancer Genome Atlas (TCGA). **D** Kaplan–Meier survival probability analysis of LUAD patients with high or low IL11 expression from TCGA data. **E** Western blot analysis of IL11 protein expression in MRC-5, A549, and H1299 cells (*n* = 4 biological replicates per group). **F**, **G** Representative images and quantitative analysis of wound healing and migration assays in MRC-5, A549, and H1299 cells (*n* = 4 biological replicates per group). Normal tissues and MRC-5 cells were used as controls. *p < 0.05 compared with the control group.

To confirm the gene expression profile of IL11 in lung cancer, we initially retrieved a meta-analysis from the Lung Cancer Explorer (LCE), an online database specific to lung cancer, to investigate the expression profile of IL11 in normal and lung cancer tissues. Analysis of lung cancer databases (LCE, TCGA) revealed significantly elevated IL11 expression in LUAD compared to normal tissues, which correlated with lymphatic metastasis and poor patient survival (Fig. 1A–D). In vitro, IL11 was highly expressed in lung cancer cell lines and associated with increased cell motility versus normal cells (Fig. 1E–G). These findings underscore substantial expression of IL11 in lung cancer and its association with metastasis and patient prognosis, highlighting its pivotal role in lung cancer progression.

### IL11 promotes migration in lung adenocarcinoma cells

**Fig. 2:**
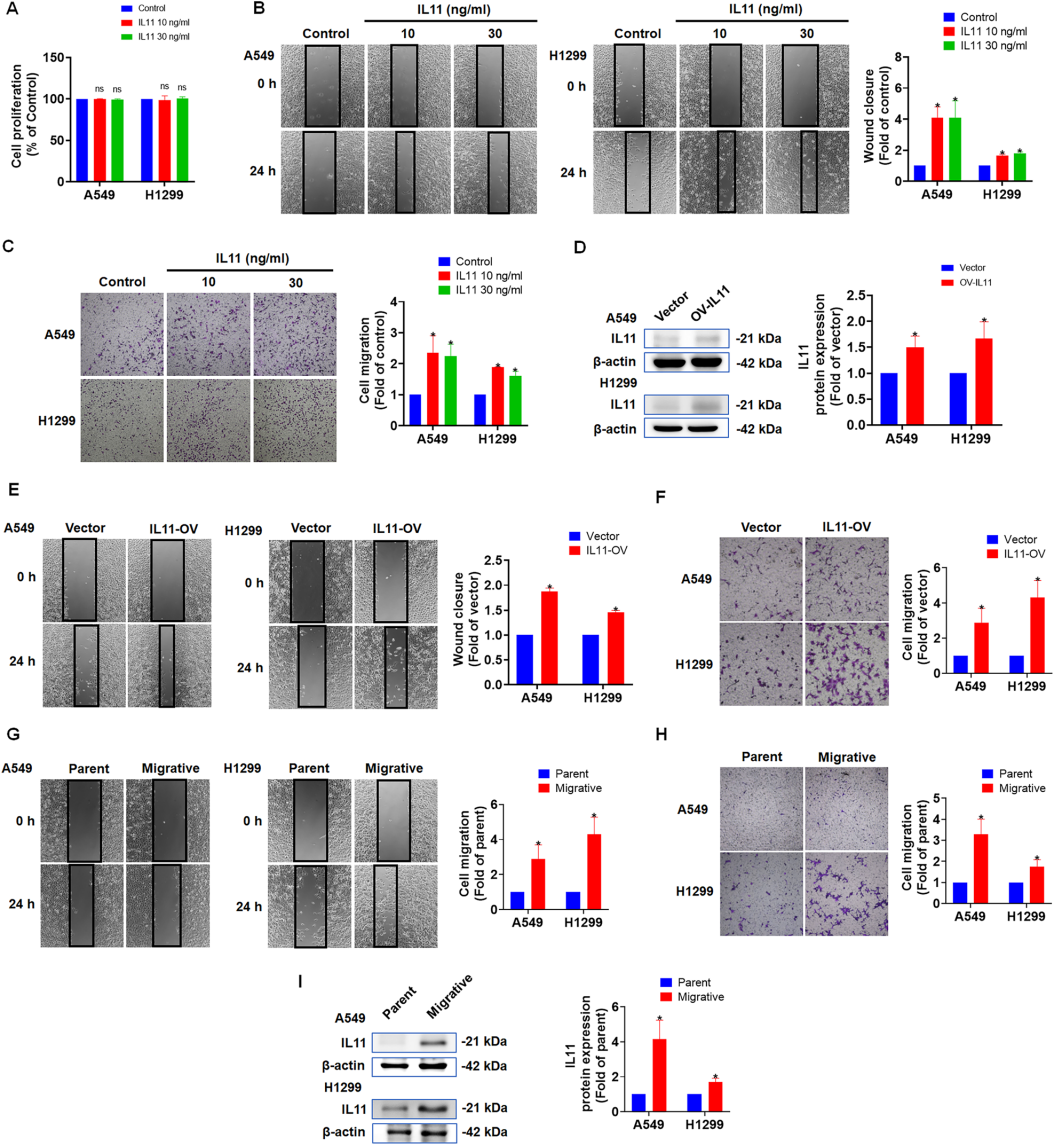
IL11 promotes migration in NSCLC cells. **A** Cell viability of NSCLC cells following IL-11 treatment for 24 h (*n* = 4 biological replicates per group). **B**, **C** Representative images and quantification of wound healing and migration assays in A549 and H1299 cells treated with IL11 (0, 10, and 30 ng/ml) for 24 h (*n* = 4 biological replicates per group). **D** Western blot analysis of IL-11 protein expression after transfection with IL11 Human Tagged Lenti ORF Clone (*n* = 4 biological replicates per group). **E**, **F** Wound healing and migration assays of A549 and H1299 cells stably overexpressing IL11 (*n* = 4 biological replicates per group). **G**, **H** Migration assays used to screen highly motile NSCLC cells, followed by wound healing and migration assays to compare motility between parental and highly migratory NSCLC cells (*n* = 4 biological replicates per group). **I** Western blot analysis of IL11 protein expression in parental and migratory NSCLC cells (*n* = 4 biological replicates per group). Untreated cells, empty vector, and parental cells were used as controls. *p < 0.05 compared with the control group.

Building upon our initial findings, we further investigated the role of IL11 in lung cancer cell metastasis. IL11 treatment enhanced cell motility in a dose-dependent manner without affecting proliferation (Fig. 2A–C). To further validate the crucial role of IL11 in lung cancer metastasis, we generated stable IL11-overexpressing A549 cell lines (IL11-OV), as demonstrated in Fig. 2D. The overexpression of IL11 significantly augmented cell mobility compared to vector control cells (Fig. 2E, F). Furthermore, we established highly migratory cell lines (A549-migrative and H1299-migrative) through migration assays (Fig. 2G, H). Subsequent western blot analyses revealed a clear association between elevated IL11 protein levels and increased cell motility in these highly migrative lung cancer cell populations (Fig. 2I). These results support IL11’s function in enhancing metastatic potential.

### IL11 promotes migration via inducing MMP12 expression in LUAD

**Fig. 3:**
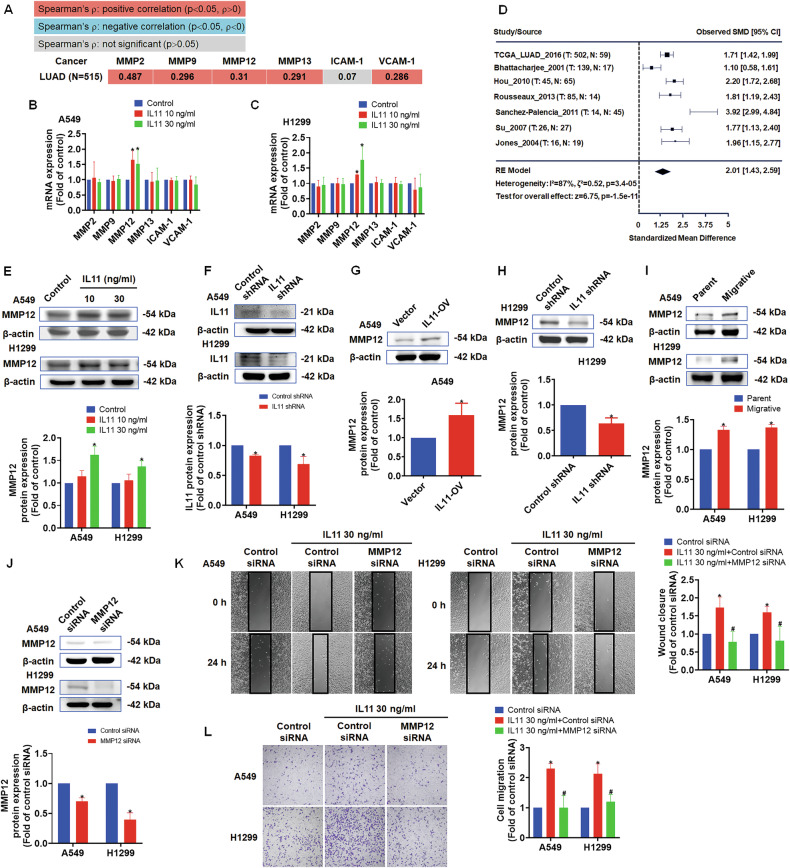
IL11 is positively correlated with MMP12 in lung cancer. **A** Correlation between MMPs, CAMs, and IL11 in LUAD from TIMER2.0. **B**, **C** qPCR analysis of MMP and CAM expression after IL11 induction for 24 h (*n* = 4 biological replicates per group). **D** Meta-analysis of MMP12 expression in normal and LUAD tissues using LCE. **E** Western blot analysis of MMP12 protein expression after treating A549 and H1299 cells with IL11 (0, 10, and 30 ng/ml) for 24 h (*n* = 4 biological replicates per group). **F** Western blot analysis of IL-11 protein expression after IL11 shRNA transfection (n = 4 biological replicates per group). **G**, **H** Western blot analysis of MMP12 expression in IL11-overexpressing A549 cells and IL11 knockdown H1299 cells (*n* = 4 biological replicates per group). **I** Western blot analysis of MMP12 expression in parental and migratory NSCLC cells (*n* = 4 biological replicates per group). **J** Western blot analysis of MMP12 expression following MMP12 siRNA transfection in NSCLC cells (*n* = 4 biological replicates per group). **K**, **L** Representative images and quantification of wound healing and migration assays in NSCLC cells treated with IL11 (0 and 30 ng/ml) for 24 h following MMP12 siRNA transfection (*n* = 4 biological replicates per group). Untreated cells, empty vector, control shRNA, control siRNA, and parental cells were used as controls. *p < 0.05 compared with the control group; # *p* < 0.05 compared with the IL11-treated group.

Previous studies have shown that the expression of matrix metalloproteinases (MMPs) is related to cancer progression and may even contribute to the deterioration of lung cancer [10]. First, we explored the association between IL11 expression and MMP activity in lung adenocarcinoma (LUAD). Bioinformatic analyses showed positive correlations between IL11 and several MMPs in LUAD (Fig. 3A), but qPCR and western blot confirmed that only MMP12 was upregulated by IL11 (Fig. 3B, C, E). Interestingly, either stable overexpression or knockdown of IL11 resulted in corresponding changes in MMP12 expression levels (Fig. 3F–H), reinforcing the direct regulatory relationship between IL11 and MMP12 in lung cancer cells. Furthermore, we observed elevated MMP12 expression in cells exhibiting greater migratory capacity (Fig. 3I). Subsequent functional analyses showed that silencing MMP12 effectively reduced IL11-induced cellular motility (Fig. 3J–L), indicating IL11 enhances migration via MMP12 induction.

### IL11 promotes migration and MMP12 expression via IL11RA/IL6ST

**Fig. 4:**
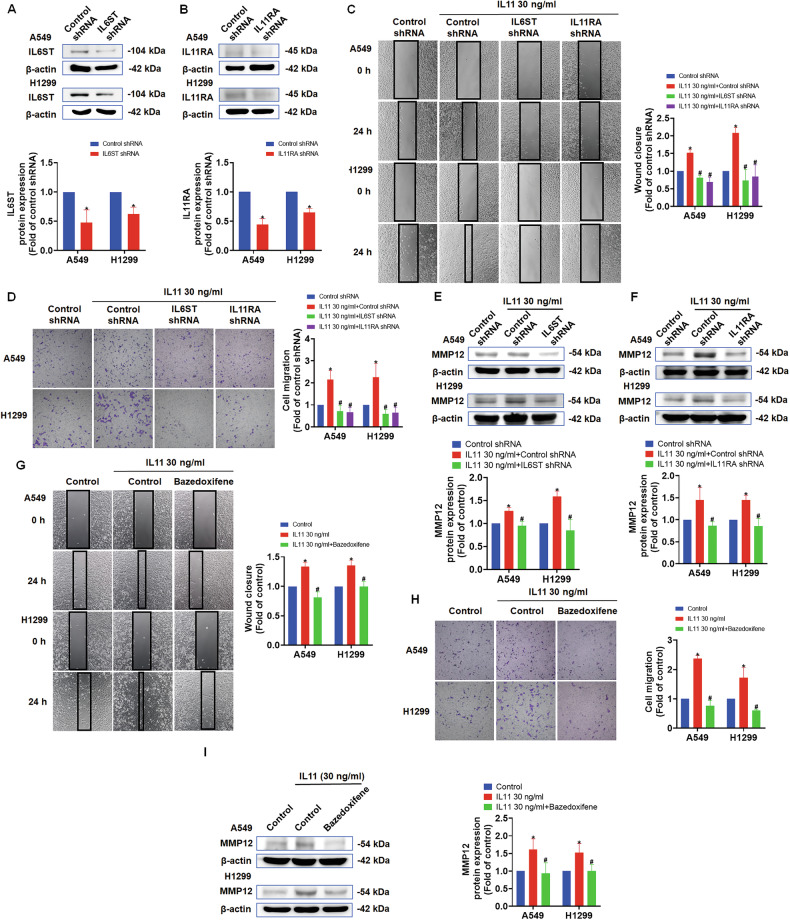
IL11 induces cancer cell migration through IL11RA/IL6ST. **A**, **B** Western blot analysis of IL6ST and IL11RA expression after transfection with control or specific shRNA in NSCLC cells (*n* = 4 biological replicates per group). **C**, **D** Representative images and quantification of wound healing and migration assays in NSCLC cells treated with IL11 (0 and 30 ng/ml) for 24 h following IL11RA or IL6ST knockdown (*n* = 4 biological replicates per group). **E**, **F** Western blot analysis of MMP12 expression in NSCLC cells treated with IL11 (0 and 30 ng/ml) for 24 h following IL11RA or IL6ST knockdown (*n* = 4 biological replicates per group). **G**, **H** Representative images and quantification of wound healing and migration assays in NSCLC cells treated with IL11 (0 and 30 ng/ml) for 24 h following bazedoxifene treatment (*n* = 4 biological replicates per group). **I** Western blot analysis of MMP12 expression following bazedoxifene treatment (*n* = 4 biological replicates per group). Control shRNA and untreated cells were used as controls. * *p* < 0.05 compared with the control group; # *p* < 0.05 compared with the IL11-treated group.

A previous study has established that IL11 signals through two receptors, IL11RA and IL6ST, mediate downstream biological effects [31, 32]. To confirm that this signaling axis is also operational in lung cancer, we knocked down IL11RA and IL6ST expression using specific shRNA in lung cancer cell lines, verifying effective silencing by protein analysis (Fig. 4A, B). We then assessed the functional role of IL11RA and IL6ST in IL11-driven cell migration through wound healing and transwell migration assays. Our results showed that decreased expression of either IL11RA or IL6ST significantly attenuated IL11-induced cell motility and reduced MMP12 protein levels (Fig. 4C–F). Additionally, pharmacological inhibition of IL6ST signaling using the specific bazedoxifene before IL11 treatment similarly suppressed IL11-mediated enhancement of cell motility (Fig. 4G, H). Correspondingly, pre-treatment with bazedoxifene significantly reduced IL11-induced MMP12 protein expression (Fig. 4I). These findings collectively support the critical role of IL11 signaling via IL11RA and IL6ST receptors in promoting lung cancer cell MMP12 expression and metastasis.

### IL11 promotes migration and MMP12 expression via IL11RA/IL6ST-dependent PI3K/Akt pathway

**Fig. 5:**
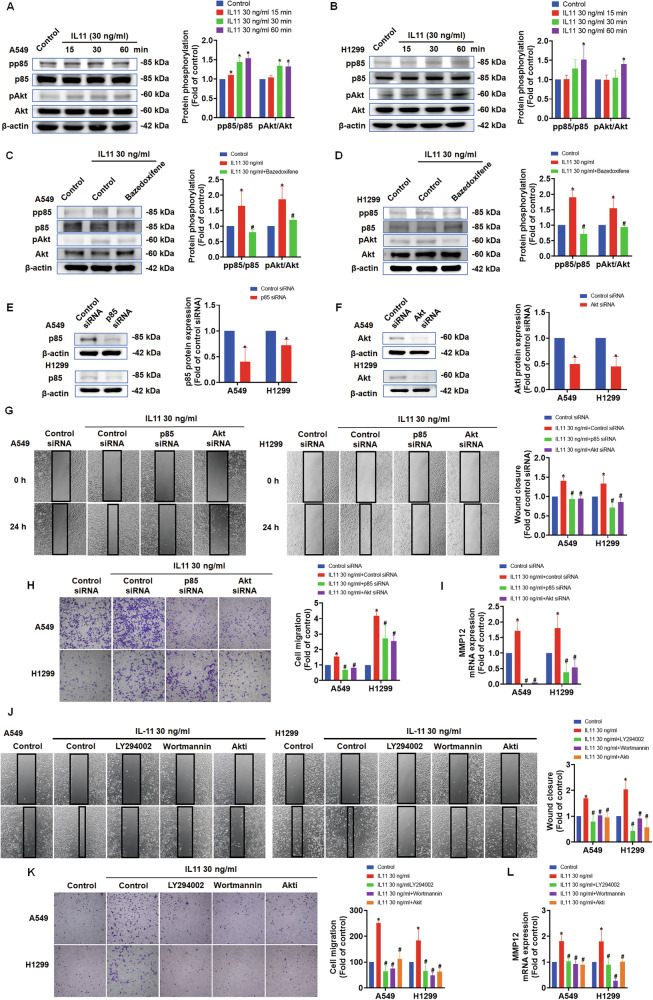
IL11 promotes motility in NSCLC cells through the PI3K/Akt pathway. **A**, **B** Western blot analysis of PI3K/Akt phosphorylation after IL11 (30 ng/ml) treatment for 0-60 min in NSCLC cells (*n* = 4 biological replicates per group). **C**, **D** Western blot analysis of PI3K/Akt phosphorylation after IL11 treatment following bazedoxifene pretreatment (*n* = 4 biological replicates per group). **E**, **F** Western blot analysis of PI3K and Akt phosphorylation following siRNA transfection (*n* = 4 biological replicates per group). **G**, **H** Representative images and quantification of wound healing and migration assays in NSCLC cells treated with IL11 (0 and 30 ng/ml) for 24 h following PI3K or Akt knockdown (*n* = 4 biological replicates per group). **I** qPCR analysis of MMP12 expression in NSCLC cells treated with IL11 and PI3K/Akt knockdown (*n* = 4 biological replicates per group). **J**–**L** Representative images and quantification of wound healing, migration assays, and qPCR analysis of MMP12 in NSCLC cells treated with IL11 and PI3K/Akt inhibitors (LY294002, Wortmannin, and Akti) (*n* = 4 biological replicates per group). Control siRNA and untreated cells were used as controls. *p < 0.05 compared with the control group; #*p* < 0.05 compared with the IL11-treated group.

We next investigated the mechanistic role of the PI3K/Akt signaling pathway, recognized for its critical involvement in cancer metastasis, in IL11-mediated effects in NSCLC. Notably, IL11 stimulation of this pathway also imparts radioresistance in cervical cancer cells [33]. Our study delved into the PI3K/Akt pathway’s engagement in metastasis driven by the IL11/IL11RA/IL6ST axis. Western blot analyses demonstrated that IL11 induced time-dependent phosphorylation of PI3K (p85) and Akt in lung cancer cells (Fig. 5A, B). Pretreatment with the IL6ST inhibitor bazedoxifene significantly attenuated IL11-induced PI3K and Akt phosphorylation, highlighting the essential role of the IL11RA/IL6ST receptor complex in mediating IL11-driven PI3K/Akt activation (Fig. 5C, D). Furthermore, knockdown experiments using siRNAs targeting p85 or Akt demonstrated effective reduction in their respective protein levels (Fig. 5E, F). Importantly, knockdown of PI3K or Akt significantly impaired IL11-stimulated cell migration and wound closure, as demonstrated by wound healing and transwell migration assays (Fig. 5G, H). Additionally, pharmacological inhibition of PI3K and Akt signaling using specific inhibitors (PI3K inhibitor LY294002, Wortamnnin and Akt inhibitor Akti) effectively diminished IL11-mediated enhancement of MMP12 expression and cellular motility (Fig. 5J–L). These results demonstrate IL11 promotes cell migration and metastatic potential via IL11RA/IL6ST-dependent activation of the PI3K/Akt signaling pathway, subsequently elevating MMP12 expression and facilitating metastatic progression.

### IL11 initiates MMP12 expression and cell migration by activating nuclear factor-κB transcriptional activity via the PI3K/Akt pathways

**Fig. 6:**
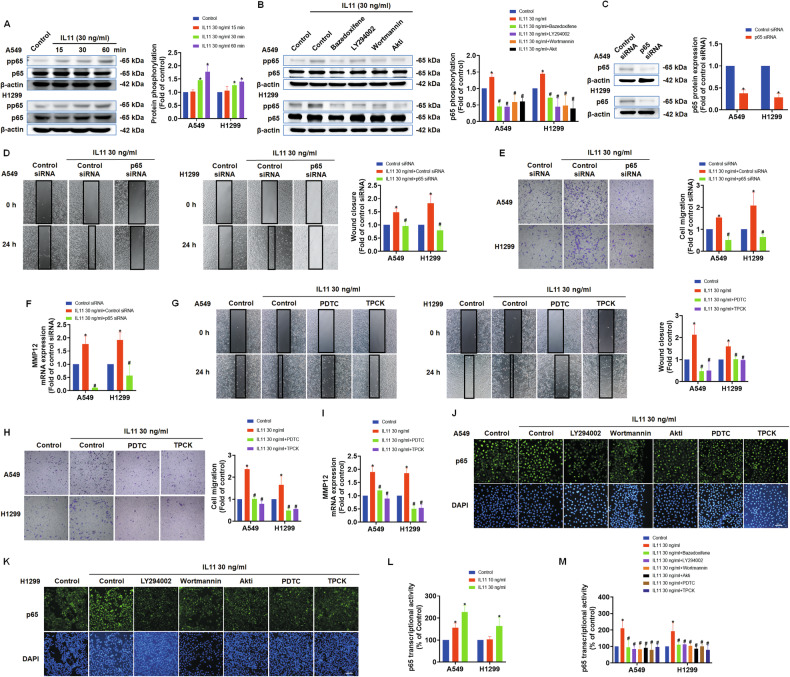
IL11 promotes motility in NSCLC cells through the PI3K/Akt-dependent NF-κB pathway. **A**, **B** Western blot analysis of p65 phosphorylation after IL11 treatment (0–60 min) and bazedoxifene, PI3K, and Akt inhibitor treatments (*n* = 4 biological replicates per group). **C** Western blot analysis of IL-11 protein expression following p65 siRNA transfection (*n* = 4 biological replicates per group). **D**, **E** Representative images and quantification of wound healing and migration assays in NSCLC cells treated with IL11 following p65 knockdown (*n* = 4 biological replicates per group). **F** qPCR analysis of MMP12 expression in IL11-treated NSCLC cells following p65 knockdown (*n* = 4 biological replicates per group). **G**–**I** Representative images and quantification of wound healing, migration assays, and qPCR analysis of MMP12 in NSCLC cells treated with IL11 and p65 inhibitors (PDTC, TPCK) (*n* = 4 biological replicates per group). **J**, **K** Immunofluorescence staining showing the intracellular distribution of p65 in IL11-treated NSCLC cells following pathway inhibitor treatments (*n* = 4 biological replicates per group). **L**, **M** Luciferase reporter assay assessing p65 transcriptional activity in IL11-treated NSCLC cells following pathway inhibition (*n* = 4 biological replicates per group). Control siRNA and untreated cells were used as controls. * *p* < 0.05 compared with the control group; # *p* < 0.05 compared with the IL11-treated group.

NF-κB, a key transcription factor in cancer progression and metastasis [34], is implicated in cancer metastasis via the PI3K/Akt/NF-κB signaling pathway [35–37]. To investigate whether IL11-induced MMP12 expression and metastasis involve NF-κB activation, we examined NF-κB signaling in our NSCLC model. Treatment with IL11 significantly enhanced phosphorylation of NF-κB p65, a hallmark of its activation, in a time-dependent manner (Fig. 6A). Pre-treatment with PI3K, Akt, and IL6ST inhibitors (LY294002, wortmannin, Akti, and bazedoxifene) significantly curtailed IL11-induced p65 phosphorylation (Fig. 6B). Further validation was obtained using p65-specific siRNA, which effectively reversed IL11-induced cell motility and reduced MMP12 mRNA expression levels (Fig. 6C–F). Similarly, p65 inhibitors (PDTC and TPCK) substantially reduced IL11-driven lung cancer cell mobility and MMP12 expression (Fig. 6G–I).

As NF-κB p65 undergoes nuclear localization upon activation, we examined the intracellular distribution of p65 by immunofluorescence. In control cells, p65 fluorescence was predominantly distributed in the cytoplasm, although the cytoplasmic signal was relatively weak. IL11 treatment increased nuclear accumulation of p65, whereas this effect was attenuated by inhibitors targeting PI3K (LY294002 and wortmannin), Akt (Akti), and NF-κB(PDTC and TPCK), indicating that these signaling molecules are required for IL11-induced NF-κB activation (Fig. 6J, K). Finally, a luciferase-based NF-κB promoter activity assay confirmed increased transcriptional activation by IL11, which was significantly attenuated by pretreatment with gp130 and PI3K/Akt inhibitors (Fig. 6L, M). These findings collectively indicate that the IL11RA/IL6ST/PI3K/Akt/NF-κB signaling pathway is crucial in IL11-induced MMP12 expression and metastasis in lung cancer cells.

### IL11 knockdown suppresses orthotopic lung tumor progression and intrapulmonary spread in vivo

**Fig. 7:**
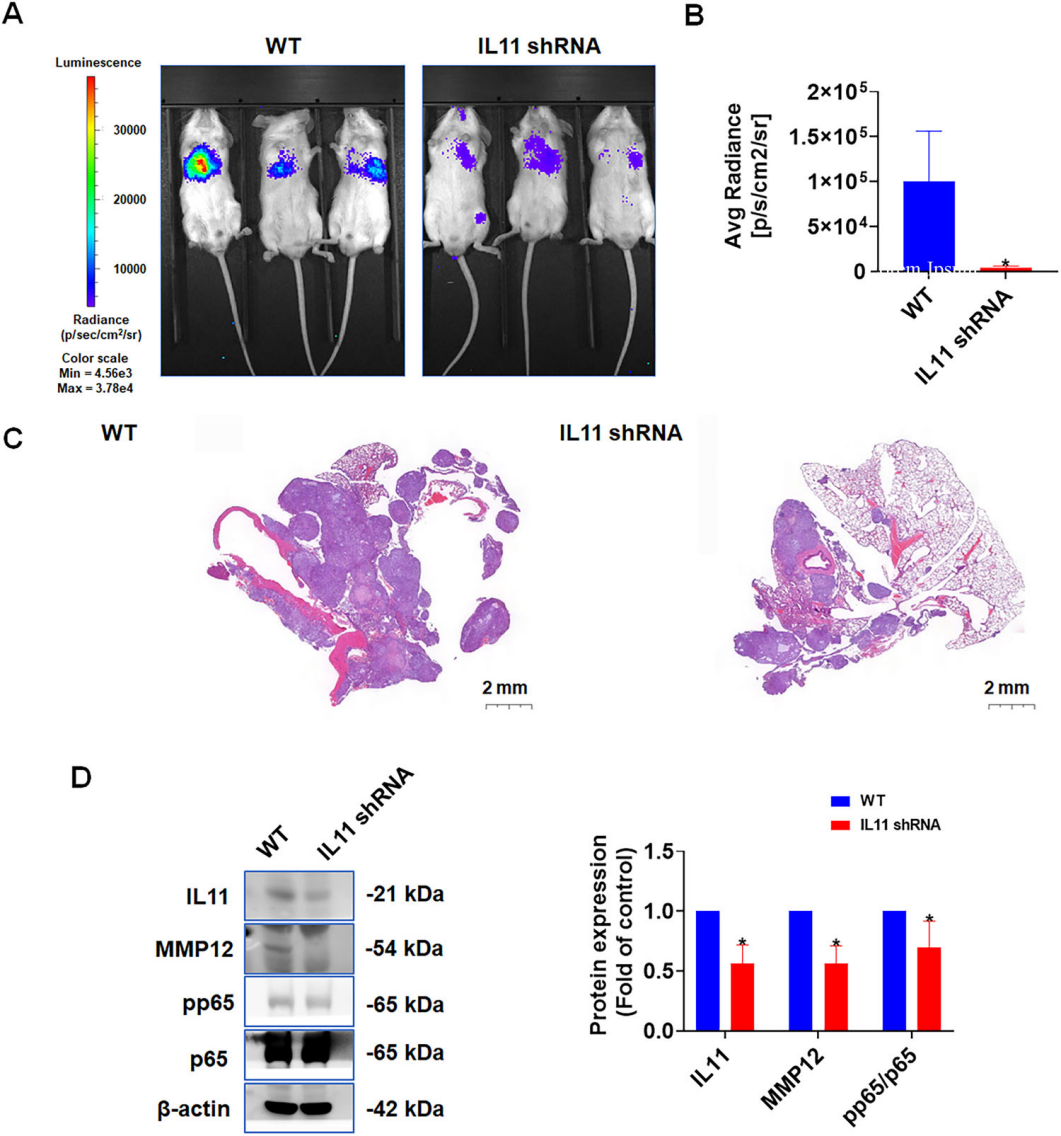
IL11 knockdown suppresses orthotopic lung tumor progression and intrapulmonary spread in vivo. **A** Representative bioluminescence images of NOD/SCID mice orthotopically implanted with luciferase-expressing H1299 cells (WT or IL11 shRNA). Tumor growth and distribution were monitored four weeks after implantation using a Revvity IVIS Lumina S5 imaging system. **B** Quantification of bioluminescent signals shown as average radiance (p/s/cm²/sr) in mice bearing WT or IL11 shRNA tumors (*n* = 6 biological replicates per group). **C** Representative hematoxylin and eosin (H&E) staining of lung sections from mice implanted with WT or IL11 shRNA cells. Scale bar = 2 mm. **D** Representative immunoblot analysis of IL11, MMP12, phosphorylated NF-κB p65 (p-p65), total p65, and β-actin in lung tissue lysates from WT and IL11 shRNA tumor-bearing mice. Lung tissue samples were analyzed as paired WT and IL11 shRNA samples on the same gel in each independent experiment (*n* = 4 biological replicates per group). WT served as the control group. *p < 0.05 compared with the WT group.

To validate our in vitro findings in vivo, we established luciferase-expressing H1299 stable cell lines carrying either wild-type control cells (WT) or IL11 shRNA. These cells were orthotopically injected into the left lung of NOD/SCID mice, and tumor growth and distribution were monitored noninvasively four weeks after implantation using an IVIS imaging system. As shown in Fig. 7A, mice implanted with WT cells exhibited stronger and more widely distributed bioluminescent signals than those implanted with IL11 shRNA cells. Quantitative analysis further showed that the average radiance was significantly higher in the WT group than in the IL11 shRNA group (Fig. 7B). To further assess tumor burden and tissue architecture, total lung tissues were collected for histological examination. H&E staining revealed that the lungs of mice in the WT group were extensively occupied by tumor lesions in both the left and right lungs, with only limited residual alveolar and bronchiolar structures observed. In contrast, in the IL11 shRNA group, tumor lesions were mainly confined to the left lung, while the right lung showed only limited nodular lesions and relatively preserved pulmonary architecture, including more intact alveolar and bronchial structures (Fig. 7C). We next examined the expression of metastasis-related proteins in lung tissues by immunoblotting. Consistent with our in vitro observations, the protein levels of IL11 and MMP12 were markedly reduced in the IL11 shRNA group compared with the WT group. In addition, NF-κB p65 phosphorylation was also decreased in the IL11 shRNA group (Fig. 7D). Together, these results support our in vitro findings and suggest that IL11 contributes to lung tumor progression and intrapulmonary spread, at least in part, through NF-κB activation and MMP12 upregulation.

## Discussion

Lung cancer, particularly NSCLC, remains a leading cause of cancer mortality and is frequently diagnosed at metastatic stages, reducing the effectiveness of conventional treatments [1, 3–6]. Consequently, identifying key drivers of metastasis and developing targeted interventions are crucial for improving therapeutic strategies. IL11, a member of the IL-6 cytokine family, is known for its roles in inflammation and tissue repair but has also been implicated in the progression and metastasis of various cancers, including breast, pancreatic, and gastric cancers [12, 15–17]. Recent reviews further emphasize that IL-11 functions as an important regulator across pulmonary inflammation, fibrosis, and cancer-associated remodeling, underscoring its relevance in lung disease progression [38]. In this study, we confirmed from public databases that IL11 is significantly upregulated in NSCLC and is associated with lymphatic metastatic progression (Fig. 1A–C). Interestingly, consistent findings were also observed through in vitro Western blot analysis (Fig. 1F–H). Despite previous reports linking IL11 to various cancers, its precise role in regulating NSCLC metastasis had not been fully elucidated. Our findings demonstrate that IL11 activates the PI3K/Akt signaling pathway via IL11RA/IL6ST, leading to NF-κB transcriptional activation and MMP12 upregulation, thereby enhancing lung cancer cell migration. Notably, we found that bazedoxifene, a clinically approved selective estrogen receptor modulator, effectively reversed IL11-induced signaling and reduced lung cancer cell motility in vitro. In addition, IL11 knockdown suppressed orthotopic lung tumor progression and intrapulmonary spread in vivo. These findings provide novel insights into the mechanism by which IL11 drives NSCLC progression and support the therapeutic potential of targeting IL11-associated signaling.

MMPs are a type of zinc-containing endopeptidases. Under physiological conditions, MMPs are primarily involved in embryogenesis, immune response, tissue remodeling, and wound healing. Under pathological conditions, MMPs can contribute to the development of fibrotic diseases, lung diseases, and cancer [39]. MMP12 plays a significant role in cancer metastasis by degrading extracellular matrix components, thereby facilitating tumor invasion and dissemination. Elevated MMP12 expression has been linked to increased metastatic potential and poor prognosis in various cancers [40–42]. For instance, in LUAD, MMP12 upregulation correlates with local recurrence and metastasis [40]. Similarly, in gastric cancer, MMP12 is associated with enhanced cell proliferation and invasion [41]. These findings underscore MMP12’s significance as a potential biomarker and therapeutic target in cancer metastasis. Analysis of public datasets showed that MMP12 is highly expressed in LUAD (Fig. 3A, D). Our experimental results are consistent with published data indicating that MMP12 production is associated with the invasiveness of lung cancer cells (Fig. 3I–L). Additionally, the increase in intracellular IL11 levels results in elevated MMP12 expression (Fig. 3B, C, E). Our data also confirmed the MMP12 depends on the IL11 level in lung cancer cells (Fig. 3G, H). Therefore, this study confirmed that IL11 enhances the migration ability of lung cancer cells by inducing the expression of MMP12.

IL11, upon binding to its specific receptor subunit IL11RA, forms a complex with IL6ST, initiating key intracellular signaling cascades that regulate various physiological processes, including hematopoiesis, bone remodeling, and inflammation. In cancer, dysregulated IL11 signaling has been implicated in tumor progression and metastasis across multiple malignancies [15–17]. However, the precise mechanisms through which IL11 promotes lung cancer metastasis remain to be fully elucidated. Our study highlights the pivotal role of the IL11RA/IL6ST receptor complex in IL11-induced NSCLC metastasis. We demonstrate that knockdown of IL11RA/IL6ST via shRNA transfection or pharmacological inhibition of IL6ST with bazedoxifene significantly reduces IL11-induced migration and MMP12 expression (Fig. 4). These findings establish that IL11RA/IL6ST is indispensable for IL11-driven metastatic behavior in lung cancer cells. Targeting this receptor complex may offer a promising therapeutic strategy to impede IL11-induced metastasis in NSCLC. Among the three major downstream pathways of IL11 signaling, JAK/STAT3, Ras/Raf/MAPK, and PI3K/Akt, the PI3K/Akt pathway has been particularly associated with the progression of various cancers [32]. IL11-mediated PI3K/Akt activation has been reported to promote cancer cell proliferation, inhibit apoptosis, and upregulate MMP expression, driving metastasis in cervical and oral cancers [33, 43]. Consistent with these findings, our study confirms that IL11 activates the PI3K/Akt pathway in NSCLC, as evidenced by increased phosphorylation levels upon IL11 treatment (Fig. 5A, B). Importantly, bazedoxifene effectively blocked IL11-induced PI3K/Akt activation (Fig. 5C, D), demonstrating that IL11 exerts its effects through IL11RA/IL6ST-dependent PI3K/Akt signaling. This activation induced MMP12 expression and enhanced cell migration (Fig. 5G–L), further reinforcing the critical role of this pathway in IL11-driven lung cancer metastasis. In addition, the Akt/NF-κB signaling axis plays a well-documented role in metastasis across multiple cancers [44–46]. The activation of Akt and NF-κB has been linked to MMP12 production, contributing to the malignancy of hepatocellular carcinoma and LUAD [47, 48]. Our findings support this mechanism, as IL11 activation of PI3K/Akt also led to NF-κB transcriptional activation, which subsequently increased MMP12 expression and cell motility. This study establishes PI3K/Akt/NF-κB as a critical downstream axis of IL11 signaling in lung cancer metastasis, offering novel insights into the mechanistic basis of IL11-induced tumor progression. To further validate the biological relevance of our in vitro findings, we employed an orthotopic lung cancer model using luciferase-labeled H1299 cells with stable IL11 knockdown. In this model, suppression of IL11 reduced bioluminescent tumor signals and limited tumor involvement in the contralateral lung. Histological analysis further showed that IL11 knockdown preserved pulmonary architecture and reduced tumor burden. Moreover, decreased IL11 expression was accompanied by reduced MMP12 expression and lower NF-κB p65 phosphorylation in lung tissues. These in vivo findings support the notion that IL11 contributes to lung tumor progression and intrapulmonary spread, at least in part, through NF-κB activation and MMP12 upregulation. Collectively, our findings highlight IL11RA/IL6ST as a central mediator of IL11-driven metastatic behavior in NSCLC, consistent with emerging evidence supporting a pathogenic role of the IL-11/IL-11RA axis in lung adenocarcinoma [26]. Our results also identify bazedoxifene as a potential therapeutic agent capable of disrupting this oncogenic signaling cascade. These insights provide a strong foundation for future clinical investigations exploring IL11-targeted interventions in lung cancer treatment.

Bazedoxifene, a selective estrogen receptor modulator approved for osteoporosis treatment, has recently been shown to inhibit IL-6/IL6ST signaling and exert anticancer effects in several malignancies [49, 50]. Our study extends these observations in NSCLC by showing that bazedoxifene suppresses IL11-induced PI3K/Akt/NF-κB signaling, reduces MMP12 expression, and attenuates cancer cell motility in vitro. In parallel, our orthotopic mouse model demonstrated that IL11 knockdown reduced tumor burden and intrapulmonary spread, accompanied by decreased IL11, MMP12, and phosphorylated NF-κB p65 levels in lung tissues. Together, these findings support a functional role for IL11-associated signaling in NSCLC progression and suggest that targeting this pathway may have therapeutic value.

In conclusion, IL11 promotes NSCLC metastatic behavior through PI3K/Akt/NF-κB-driven MMP12 upregulation. Bazedoxifene effectively disrupts this signaling axis and suppresses IL11-induced cell motility in vitro, whereas IL11 knockdown inhibits orthotopic lung tumor progression and intrapulmonary spread in vivo. These findings provide a mechanistic basis for IL11-targeted interventions and support further exploration of IL11-associated signaling as a therapeutic target in NSCLC. These conclusions are in line with emerging studies indicating that the IL-11/IL-11RA axis is therapeutically actionable in cancer and may represent a viable target for antibody-based intervention [51].

## Materials and methods

### Analysis of public data sets

Publicly available datasets were analyzed to investigate the expression levels and correlations of IL11, IL11 receptor (IL11RA), and MMP12 in lung cancer. Meta-analysis datasets were obtained from the Lung Cancer Explorer (LCE) (https://lce.biohpc.swmed.edu/lungcancer/index.php#about), incorporating gene expression profiles from The Cancer Genome Atlas (TCGA) and published studies. Data from TCGA, including lung adenocarcinoma (LUAD) and lung squamous cell carcinoma (LUSC), were further analyzed using UALCAN (http://ualcan.path.uab.edu/index.html). Correlation analyses were conducted using the TIMER database (http://timer.comp-genomics.org/timer/).

### Materials

Primary antibodies specific for phospho-phosphoinositide 3-kinase (PI3K) (Ser537, #2481), PI3K (#14247), phospho-protein kinase B (Akt) (Ser473, #4060), Akt (#9272), phospho-NF-κB p65 (Ser536, #3033), and NF-κB p65 (#8242) were purchased from Cell Signaling Technology (Danvers, MA, USA). Primary antibodies specific for IL11 (GTX66720), β-actin (GTX11003), anti-rabbit polyclonal antibody (GTX213110-01), and anti-mouse monoclonal antibody (GTX300120) were purchased from GeneTex (Irvine, CA, USA). Primary antibodies specific for MMP12 (PA5-13181) and Lipofectamine™ 3000 Transfection Reagent were purchased from ThermoFisher (Waltham, MA, USA). Recombinant human IL11 (Catalog No. 200-11) was purchased from PeproTech (Rocky Hill, NJ, USA). Other reagents were obtained from Sigma-Aldrich (St. Louis, MO, USA). Concentrations of IL11 and bazedoxifene were selected based on prior literature and preliminary dose-response assessments in cancer cell lines [52–55].

### Cell lines

NSCLC cell lines (H1299) and human embryonic kidney cells (293T) were acquired from American Type Culture Collection (ATCC) (Manassas, VA, USA), while A549 and normal lung fibroblasts (MRC-5) were obtained from Bioresource Collection and Research Center (BCRC) (Hsinchu, Taiwan). Cells were cultured under standard conditions: H1299 cells were cultured in RPMI 1640 medium supplemented with 10% FBS, 100 U/mL penicillin, and 100 μg/mL streptomycin. A549 cells were cultured in Ham’s F-12 Nutrient Mixture supplemented with same supplements as above. MRC-5 cells were cultured in Eagle’s Minimum Essential Medium (EMEM) supplemented with same supplements as above. 293T cells were cultured in Dulbecco’s Modified Eagle Medium (DMEM) supplemented with same supplements as above. Cells were maintained at 37 °C in a 5% CO_2_ incubator. All cell lines were used at passage numbers below 20. STR profiling was not performed in this study. Mycoplasma testing was not performed in this study.

### Cell migration assay

Cells (2 × 10⁴ per well) were seeded in the upper chamber of Transwell inserts (8-μm pore size; Costar, New York, NY, USA). After 24 h, cells were fixed with 4% formaldehyde for 30 min, stained with 0.05% crystal violet for 45 min, and non-migratory cells were removed. Migrated cells were visualized and quantified using ImageJ 1.52a (National Institutes of Health, Bethesda, MD, USA). A highly mobile cell line was established by repeatedly collecting and reseeding migrated cells over multiple rounds.

### Wound healing assay

H1299, A549, and MRC-5 cells (2.5 × 10⁴) were seeded in Culture-Inserts (Cat. No. 80209, ibidi GmbH, Munich, Germany). After 24 h, inserts were removed, and wound closure was monitored at 0 h and 24 h under a microscope. ImageJ 1.52a was used for quantification.

### Western blot

Protein samples were separated by 8–15% SDS-PAGE, transferred to 0.45-μm PVDF membranes (Merck Millipore), and blocked with 5% non-fat milk. Membranes were incubated overnight with primary antibodies (1:1000) at 4 °C, followed by secondary antibodies (1:10,000) for 1 h at room temperature. Chemiluminescent detection was performed using ECL reagent (RPN2235, Amersham^TM^ cytiva, Washington, DC, USA) and visualized using chemiluminescence detection system (UVP ChemiDOC-It 815, Analytik Jena, Jena, SN, BRD).

### Cell viability assay

Cells (1 × 10⁴ per well) were seeded in 48-well plates and treated with different IL11 concentrations for 24 h. Cell Counting Kit-8 (CCK-8, Sigma-Aldrich) was added to each well and incubated for 4 h. The microplate was measured at absorbance 450 nm using VARIOSKAN LUX microplate reader.

### Immunofluorescence (IF)

Cells were fixed with 4% formaldehyde, permeabilized with 0.1% Triton X-100, and blocked with 3% BSA in PBS. After incubation with primary p65 antibody (1:200) overnight, cells were stained with FITC-conjugated secondary antibody and counterstained with DAPI. Images were captured using a Nikon ECLIPSE Ti microscope (NIS Elements AR 5.02.01).

### Transient transfections

PI3K, Akt, RELA (p65), MMP12, and control siRNAs were purchased from Sigma-Aldrich (St. Louis, MO, USA) and transfected using DharmaFECT 1 transfection reagent for 24 h according to the manufacturer’s instructions. The siRNA sequences used were: PI3K: 5’-CAAAGGAUUAUGCAUAAUU-3’, Akt: 5’-CCAAGCACCGCGUGACCAU-3’, RELA (p65): 5’-GGAAUCCAGUGUGUGAAGA-3’, MMP12: 5’- AGAUUGACCAGAGCCUCCAAGGUUU-3’.

### Reporter gene assay

Cells (5 × 10⁴ per well) were seeded into 24-well plates and transfected with an NF-κB promoter reporter plasmid (Promega, Madison, WI, USA) using Lipofectamine 3000 for 24 h. Cells were then treated with IL11 for another 24 h, lysed with reporter lysis buffer (E153A, Promega, Madison, WI, USA), and analyzed for luminescence using a VICTOR X2 microplate luminometer (PerkinElmer, MA, USA). Inhibitor pre-treatment (PI3K, Akt, and NF-κB inhibitors) was conducted before IL11 treatment to examine signaling pathway regulation.

### Lentivirus transduction and generation of stable cell lines

IL11 Human Tagged Lenti ORF Clone were purchased from ORiGENE (Rockville, MD, USA). Short hairpin RNA (shRNA) targeting IL11, IL11RA, IL6ST, and an eGFP-luciferase plasmid were obtained from National RNAi Core Facility Platform (Taipei, Taiwan). H1299 and A549 cells were transduced with lentiviral particles and selected using puromycin (2–10 μg/mL). Stable expressing IL11 shRNA-luciferase-GFP and overexpressing IL11-luciferase-GFP cells were established.

### Quantitative real-time polymerase chain reaction (qPCR)

Total RNA was extracted using easy-BLUE total RNA extraction kit (iNtRON Biotechnology, Korea), and cDNA was synthesized using qPCRBIO cDNA Synthesis Kit (PCR Biosystems, UK). Gene expression was analyzed via qPCR using KAPA SYBR FAST qPCR Master Mix (Sigma-Aldrich) on a CFX Connect Real-Time PCR System (BioRad, USA). GAPDH was used as the internal control. The primer sequences: IL11: Forward: 5’-GGGGACATGAACTAGGGACA-3’, Reverse: 5’-GTAGGACAGTAGGTCCGCTC-3’, MMP2: Forward: 5’-GTGATCTTGACCAGAATACC-3’, Reverse: 5’-GCCAATGATCCTGTATGTG-3’, MMP9: Forward: 5’-AGCTTGCAGAGGAATAC-3’, Reverse: 5’-CCCCAGAGATTTCGACTC-3’, MMP12: Forward: 5’-AGTTTTGATGCTGTCACTAC-3’, Reverse: 5’-TTCATAAGCAGCTTCAATGC-3’, MMP13: Forward: 5’-AGGTAGAAAATTTTGGGCTC-3’, Reverse: 5’-CTTAACTTCTTTTGGAAGACCC-3’, ICAM-1: Forward: 5’-ACCATCTACAGCTTTCCG-3’, Reverse: 5’-TCACACTTCACTGTCACC-3’, VCAM-1: Forward: 5’-TGGGAAGATGGTCGTGATCC-3’, Reverse: 5’-TCGTCACCTTCCCATTCAGT-3’, GAPDH: Forward: 5’-ACAGTGCATGTAGACC-3’, Reverse: 5’-TTGAGCACAGGGTACTTA-3’.

### In vivo orthotopic lung cancer model

An orthotopic lung cancer model was established as previously described with minor modifications [56, 57]. Briefly, twelve 5-week-old male NOD/SCID mice were anesthetized with 2% isoflurane and randomly assigned into two groups (*n* = 6 per group). H1299 cells (WT or IL11 shRNA; 5 × 10^5^ cells) were resuspended in 50 μL of a 1:1 mixture of RPMI and Matrigel and orthotopically injected into the left lung of each mouse. Four weeks after implantation, tumor growth was assessed using a Revvity IVIS Lumina S5 imaging system (Revvity, Waltham, MA, USA). Mice were subsequently euthanized, and both lungs were harvested for further analyses. Lung tissues were fixed in 4% paraformaldehyde, embedded in paraffin, and sectioned for hematoxylin and eosin (H&E) staining. For mechanistic analysis, protein lysates prepared from lung tissues were subjected to immunoblotting to determine the expression of IL11, MMP12, and the phosphorylation of NF-κB. All animal experiments were conducted in accordance with protocols approved by the Institutional Animal Care and Use Committee of Shin Kong Wu Ho-Su Memorial Hospital (IACUC Approval No. 112NSTCIACUC010).

### Statistical analysis

Sample sizes for in vitro experiments were selected based on prior experience, previous related studies, and the feasibility of the experimental assays. For the in vivo orthotopic lung cancer model, the group size was determined based on prior experience with this model and practical considerations for detecting biologically meaningful differences between groups. No formal power calculation was performed. All in vitro experiments were independently repeated at least three times, and representative data from biological replicates are shown in the figures as indicated in the figure legends. No samples or animals were excluded from the analyses, and no pre-established exclusion criteria were applied in this study. For the animal study, mice were randomly assigned to experimental groups. No blinding was performed during group allocation, data collection, or outcome assessment. Data are presented as mean ± standard deviation (SD) and were analyzed under the assumption of approximate normal distribution and comparable variance between groups for parametric testing. Statistical analyses were performed using GraphPad Prism 8.0 (GraphPad Software, San Diego, CA, USA). Comparisons between two groups were performed using Student’s *t* test. For comparisons among multiple groups, one-way ANOVA followed by Tukey’s post hoc test was used. For experiments involving two independent variables, two-way ANOVA followed by Sidak’s post hoc test was performed, as appropriate. A *p* value < 0.05 was considered statistically significant. Statistical significance is indicated in the figures as * or # for *p* < 0.05.

#### Ethical approval

All animal experiments were performed in accordance with the relevant guidelines and regulations and were approved by the Institutional Animal Care and Use Committee of Shin Kong Wu Ho-Su Memorial Hospital (IACUC Approval No. 112NSTCIACUC010). Animal handling and reporting were conducted in accordance with the ARRIVE guidelines, where applicable. This study did not involve human participants, human tissue, or identifiable human data; therefore, informed consent was not applicable.

## Supplementary information

Supplementary information

## Data Availability

All other data needed to evaluate the conclusions in the paper are present in the paper or the Supplementary Materials. The datasets generated for this study can be accessed upon request to the corresponding author.

## Code availability

No custom computer code central to the conclusions of this study was generated. Publicly accessible online tools and standard commercial software were used for data analysis and statistics, including LCE, UALCAN, TIMER, GraphPad Prism 8.0, and ImageJ 1.52a.
