# Supplementary material for: IL11 modulates MMP12 expression and cancer cell metastasis via IL11RA/IL6ST-PI3K/Akt/NF-κB pathway in lung cancer
Source: Cell Death Discov. 2026 Jun 11;12:337. doi: 10.1038/s41420-026-03163-2 (PMC13478155; doi:10.1038/s41420-026-03163-2)
Supplement: Supplementary file 1 [file 41420_2026_3163_MOESM1_ESM.pdf]

Supplementary Information

The previously provided Supplementary Figure has been removed because all four panels have been incorporated into the corresponding main figures of the article. No additional supplementary data remain.
